# Incidentally Detected Basal Ganglia Calcifications Are Not Associated with Impaired Mobility and Recurrent Falls in Older Adults

**DOI:** 10.3390/jcm15124732

**Published:** 2026-06-18

**Authors:** Irene M. de Graaf, Annemarieke de Jonghe, Nienke M. S. Golüke, Esther J. M. de Brouwer, Mariëlle H. Emmelot-Vonk, Pim A. de Jong, Lydia C. M. Kwekkeboom, Huiberdina L. Koek

**Affiliations:** 1 Department of Geriatrics, University Medical Center Utrecht, Utrecht University, Heidelberglaan 100, 3584 CX Utrecht, The Netherlandsh.l.koek@umcutrecht.nl (H.L.K.); 2 Department of Geriatrics, Tergooi MC, 1201 DA Hilversum, The Netherlands; 3 Department of Geriatrics, Hospital Gelderse Vallei, 6716 RP Ede, The Netherlands; 4 Department of Geriatrics, Deventer Hospitali, 7416 SE Deventer, The Netherlands; 5 Department of Radiology, Meander MC, 3818 ES Amersfoort, The Netherlands; 6 Department of Geriatrics, Beatrix Hospital Gorinchem, 4204 AA Gorinchem, The Netherlands

**Keywords:** basal ganglia calcifications, mobility impairment, falls, older adults, computed tomography

## Abstract

**Background:** Basal ganglia calcifications (BGCs) are frequently detected on brain CT scans in older adults, but their clinical relevance for mobility and fall risk is unclear. This study investigated the association of BGCs with impaired mobility and recurrent falls. **Methods:** In this cross-sectional study, all consecutive patients referred to the mobility clinic of a regional teaching hospital between 2019 and 2021 were included. Mobility was assessed using the Performance-Oriented Mobility Assessment (POMA) for balance, gait and overall mobility, and the Timed Up and Go (TUG) test for functional mobility. All assessments were performed by a trained physiotherapist. Recurrent falls were defined as self-reported occurrence of more than one fall in the past 12 months. Brain CT scans were evaluated for BGCs by a trained senior radiologist and were scored by severity. Univariable and multivariable logistic regression analyses were performed, adjusting for age, sex, and history of cardiovascular events. **Results:** A total of 253 participants were included (median age 82 years; 58% female), of whom 31% had BGCs. Falls data were available for 246 participants, and 70% reported recurrent falls. In both univariable and multivariable analyses, there was no evidence of a statistically significant association between the presence of BGCs and impaired balance, gait, overall mobility, functional mobility, or recurrent falls. **Conclusions:** No evidence of a statistically significant association was found between incidentally detected BGCs and impaired mobility or recurrent falls in older adults. Further longitudinal research is needed to confirm these findings and clarify whether BGCs are clinically relevant for mobility and fall risk assessment.

## 1. Introduction

Mobility impairments are well known risk factors of falls in older adults [1]. Falls represent a common and significant health concern among adults over the age of 60 worldwide, with a reported global prevalence of approximately 27%. As life expectancy continues to rise, the proportion of older individuals in the population is increasing, contributing to a parallel rise in the incidence of falls [2].

Impaired mobility is one of the strongest risk factors for falls and affects approximately 35% of adults aged 70 years and older [3]. In particular, slow gait speed has been consistently associated with an increased risk of adverse outcomes, including mortality [3]. The control of movement relies on complex neural networks, in which the basal ganglia play a central role. Dysfunction of these structures may result in motor impairments, such as slowed or abnormal movements [3]. Therefore, older adults presenting with falls or mobility problems are often evaluated for underlying neurological causes, frequently including brain imaging with computed tomography (CT). During these evaluations, incidental findings are common. One of the most frequently observed findings is basal ganglia calcification (BGC), with reported prevalences of up to 40% and increasing occurrence with advancing age [4,5].

Although often regarded as an age-related finding, BGCs are also a characteristic feature of primary familial brain calcification (PFBC; formerly Fahr’s disease), a rare neurodegenerative disorder in which movement abnormalities are common [6]. This overlap creates uncertainty regarding the clinical interpretation of incidentally detected BGCs. While the calcification burden in incidental BGCs is generally lower than that observed in PFBC, the clinical relevance of these findings remains poorly understood. Consequently, incidental BGCs are often considered of limited significance and rarely prompt further evaluation. However, previous studies have reported associations between intracranial calcifications and motor dysfunction suggesting that the potential impact of BGCs on motor function may not be fully understood [7]. Clarifying whether incidentally detected BGCs are associated with impaired mobility or recurrent falls could help determine whether these findings represent a benign age-related phenomenon or warrant greater clinical attention, particularly in patients with a substantial calcification burden. Therefore, this study aimed to investigate the associations of both BGC presence and BGC severity with mobility impairments and recurrent falls in older adults.

## 2. Methods

### 2.1. Study Design

This cross-sectional study included all consecutive patients referred to the mobility clinic of Tergooi MC, a regional teaching hospital in the Netherlands, between January 2019 and December 2021. Data were collected prospectively as part of an ongoing observational study embedded in routine clinical care. To reflect routine clinical practice, no age-based inclusion criterion was applied, and all consecutive patients referred for mobility screening were considered eligible for inclusion. Only patients who lacked either a mobility assessment or a brain CT scan were excluded from the study. The study was reported in accordance with the STROBE guidelines (Strengthening the Reporting of Observational Studies in Epidemiology) [8]. According to routine clinical practice, each patient underwent a standardized assessment, which included medical history, medication review, evaluation of vital signs, laboratory testing, brain CT, and a mobility assessment. All evaluations were conducted by a dedicated mobility clinic team consisting of a geriatrician, a physiotherapist, and a nurse.

### 2.2. Clinical Variables

The following clinical data were retrieved from patient records: age, sex, living situation, cardiovascular risk factors (including hypertension, diabetes mellitus, hypercholesterolemia, renal insufficiency, and smoking), and cardiovascular comorbidities (including myocardial infarction, stroke, transient ischemic attack [TIA], and peripheral arterial disease). Hypertension, diabetes mellitus, and hypercholesterolemia were considered present if documented in the medical history or if the patient was receiving relevant pharmacological treatment. Smoking status was categorized as current smoker or non-smoker. Renal insufficiency was defined as an estimated glomerular filtration rate (eGFR) <30 mL/min/1.73 m^2^, and classified as reduced if the eGFR was between 30 and 60 mL/min/1.73 m^2^. Cardiovascular comorbidities were defined as a history of myocardial infarction, TIA, stroke, or peripheral arterial disease, or the presence of a stroke visible on brain CT.

### 2.3. Imaging Variables

Imaging of the brain was performed with a 64-detector row CT (Somatom Definition AS; Siemens Healthineers, Erlangen, Germany). All CT scans were reviewed and scored for the presence and severity of BGCs by a single trained, senior radiologist [PJ] who was blinded to the outcomes and clinical variables. Because all scans were assessed by one rater, inter-rater reliability was not evaluated. Severity was scored as follows: calcifications were considered ‘mild’ if there was 1 high-attenuation area, ‘moderate’ if there were multiple high attenuation areas and ‘severe’ if these calcifications were confluent. The left and right basal ganglia were scored separately. The side with the highest score (i.e., either left or right) was noted.

### 2.4. Outcome Variables

The outcome variables included impaired balance, gait, overall physical performance and recurrent falls. Balance and gait were evaluated using the Tinetti Performance-Oriented Mobility Assessment (POMA), which comprises two subscales: POMA-Balance (POMA-B) and POMA-Gait (POMA-G). The balance subscale evaluates performance during tasks such as sitting, standing, turning, and maintaining postural stability, whereas the gait subscale assesses gait initiation, step characteristics, symmetry, continuity, path deviation, and trunk stability during walking. The maximum score of the POMA-B and POMA-G combined is 28 (POMA-B max 16, POMA-G 12). Impaired balance was defined as a POMA-B score < 10, impaired gait as a POMA-G score < 9, and reduced overall mobility as a total POMA score < 19 [9]. Functional mobility, defined as the combined capacity of strength, mobility, and balance, was assessed using the Timed Up and Go (TUG) test. The Timed Up and Go test assesses functional mobility by measuring the time required for a participant to stand up from a chair, walk 3 m, turn around, walk back, and sit down again. Impaired functional mobility on the TUG test was defined as a completion time > 20 s [10]. Both instruments are recommended tools for evaluating mobility in older adults, according to the American Geriatrics Society/British Geriatrics Society Panel on Prevention of Falls in Older Persons [11]. Both the POMA and the TUG assessment were performed by a physiotherapist trained in the standardized assessment procedures, who also documented the results according to the study protocol. 

Furthermore, recurrent falls were defined as patient self-report of having fallen more than once in the past 12 months.

### 2.5. Data Analysis

Baseline characteristics were summarized using descriptive statistics. Binary logistic regression analyses were conducted to examine the association between the presence of BGCs and impaired mobility. Both univariable (unadjusted) and multivariable (adjusted) models were employed.

Three primary binary outcome measures were evaluated: an abnormal Timed Up and Go (TUG) test, an abnormal total Performance-Oriented Mobility Assessment (POMA) score and the presence of recurrent falls (yes/no). In addition, the POMA gait and balance subscales were analyzed separately. BGCs were graded as absent, mild, moderate, or severe. For the primary analyses, BGC severity categories were dichotomized into absent versus present, with the latter category including all degrees of calcification (mild, moderate, and severe). Multivariable models were adjusted for age (continuous), sex, and history of cardiovascular events (yes/no). The results are presented as odds ratios (ORs) with corresponding 95% confidence intervals (95% CIs). Statistical significance was defined as a two-sided *p*-value < 0.05. All statistical analyses were performed using SPSS Statistics version 31.0.0.0 (IBM Corp., Armonk, NY, USA).

### 2.6. Ethics

The study used routinely collected clinical data that were analyzed in a pseudonymized manner. The study was assessed by the local research governance office of Tergooi MC and was determined not to fall under the scope of the Dutch Medical Research Involving Human Subjects Act. Therefore, formal review by a Medical Ethics Review Committee and individual written informed consent were not required. The study was conducted in accordance with applicable institutional policies and privacy regulations.

## 3. Results

### 3.1. Cohort Description

Data of 253 patients visiting the mobility clinic was obtained. No patients were excluded because of the absence of a CT scan or mobility assessment. Baseline characteristics of the study population are presented in Table 1. In our cohort 58% were female, the median age was 82 years (range 52–95 years), and 97% were 65 years or older. In total, 45.5% of the patients had a history of a cardiovascular event. The prevalence of previous stroke appeared higher among patients with moderate BGCs (41%) than among those without BGCs (22%).

In 174 of the cases there were no BGCs (68.8%). In the case of present BGCs (*n* = 79), 17% were scored mild, 11% had moderate calcifications and only 3% had severe calcifications (7/253).

### 3.2. Outcome Measurements

The mean TUG completion time was 14.0 s (SD 7.5). The mean POMA scores were 12.1 (SD 3.2) for balance, 9.3 (SD 2.8) for gait and 21.3 (SD 5.0) for the total score. Among the 246 participants with available data, 173 (70%) reported recurrent falls (defined as more than one fall in the past 12 months). Missing falls data (*n* = 7) were infrequent (3%) and were not concentrated within a particular BGC severity category. No missing data were present for the other outcome measures, namely the Performance-Oriented Mobility Assessment (POMA) and Timed Up and Go (TUG) test.

Descriptive mobility outcomes of balance, gait, and functional and overall mobility are presented stratified by BGC severity (none, mild, moderate, and severe) (Table 2).

In univariable binary logistic regression analyses (Table 3), the presence of BGCs was not significantly associated with abnormal gait, balance, overall mobility, functional mobility, or recurrent falls. ORs ranged from 0.93 to 1.12, with no statistical significance.

After adjustment for age, sex, and history of cardiovascular events, no statistically significant associations were observed between BGC presence and any mobility outcome. Adjusted ORs were 1.16 (95% CI 0.64–2.01) for abnormal gait, 1.03 (95% CI 0.53–2.00) for abnormal balance, 1.09 (95% CI 0.59–2.03) for abnormal overall mobility, and 1.37 (95% CI 0.65–2.91) for abnormal functional mobility. The adjusted OR for recurrent falls was 0.91 (95% CI 0.50–1.66). None of the associations reached statistical significance (all *p*-values > 0.05) (Table 3). Also, sensitivity analyses using continuous POMA and TUG scores did not reveal significant associations between BGCs and mobility outcomes.

## 4. Discussion

In this study, no statistically significant associations were observed between BGC presence and mobility outcomes or recurrent falls. Mobility outcomes included balance, gait, and overall mobility assessed by the POMA, as well as functional mobility assessed by the TUG test.

Data on the functional implications of incidental BGCs are limited, because most of the available literature focuses on rare syndromic or metabolic causes of extensive calcifications rather than on incidental findings commonly observed on neuroimaging in older populations. To our knowledge, this is one of the first studies to specifically investigate the relationship between incidentally detected BGCs and objectively assessed mobility outcomes in a clinically relevant population of older adults referred for mobility assessment. The prevalence of BGC in our cohort (31.2%) was comparable to that reported in population-based studies from Sweden (38.7%) [12] and the Netherlands (29.7%) [13], supporting the representativeness of our population. Although mean mobility scores were not severely impaired (mean POMA score of 21; mean TUG score of 14 s), our cohort was clinically relevant, as these values are associated with an increased risk of falls [9]. A major strength of this study is the rigorous assessment of mobility using different validated instruments, performed by a trained physiotherapist. Both the POMA and the TUG test have demonstrated good reliability and validity in older populations [14,15].

However, this study also has limitations. While the overall cohort size was adequate for the primary analyses, the number of participants with higher-grade BGCs was limited. Therefore, the mild, moderate, and severe categories were combined into a single “BGC present” group. As a result, the study was underpowered to reliably assess dose–response relationships between increasing BGC severity and mobility impairment or recurrent falls. Furthermore, participants were recruited from a specialized mobility clinic and already presented with mobility-related complaints. Consequently, while BGCs themselves were incidental findings, the study population represents a selected clinical cohort. The findings may therefore not be fully generalizable to community-dwelling older adults with incidentally detected BGCs. Falls were assessed retrospectively based on patient self-report, which may have introduced recall bias and resulted in some misclassification of fall history. In addition, although analyses were adjusted for relevant covariates, residual confounding from unmeasured factors cannot be excluded.

Because of the cross-sectional design and the absence of longitudinal imaging and follow-up data, no conclusions can be drawn regarding causality, the temporal relationship between BGCs and mobility outcomes, or the progression of calcifications over time. Consequently, it remains unclear whether BGCs are stable findings or whether progressive calcification may be associated with subsequent mobility decline or other clinically relevant outcomes. Longitudinal studies are therefore needed to further clarify the natural history and clinical significance of BGCs.

In conclusion, no statistically significant associations were observed between incidentally detected BGCs and impaired mobility or recurrent falls in older adults referred for mobility assessment. While these findings do not support a major role for incidental BGCs in mobility impairment, the study was not powered to exclude more modest or severity-dependent associations. Future studies with larger numbers of participants with extensive calcifications and longitudinal follow-up are needed to determine whether BGCs are associated with subsequent mobility decline, fall risk, or other clinically relevant outcomes over time. Until such evidence becomes available, preventive strategies aimed at preserving mobility and reducing fall risk should continue to focus on established and modifiable risk factors, including cardiovascular risk factors.

## Figures and Tables

**Table 1:** Baseline characteristics (*n* = 253).

Patient Characteristics	Total *N* (%)	No BGC *N* (%)	Mild BGC *N* (%)	Moderate BGC *N* (%)	Severe BGC *N* (%)
Total *N* (%)	253 (100)	174 (69)	43 (17)	29 (11)	7 (3)
Female	147 (58)	99 (57)	27 (63)	17 (57)	4 (57)
Age in years (median, range)	82 (52–95)	81 (52–95)	80 (66–93)	82 (70–93)	81 (65–94)
Cardiovascular risk factors					
Hypertension	171 (68)	123 (71)	31 (72)	14 (48)	3 (43)
Diabetes mellitus (*n* = 250)	54 (22)	36 (21)	10 (24)	5 (18)	3 (43)
Hypercholesterolemia (*n* = 252)	70 (28)	47 (27)	13 (30)	9 (31)	1 (14)
Smoking (*n* = 250)	25 (10)	16 (9)	4 (9)	3 (11)	2 (29)
Cardiovascular comorbidities					
Myocardial infarction (*n* = 252)	40 (16)	30 (17)	6 (14)	3 (10)	1(14)
Stroke	65 (26)	39 (22)	12 (28)	12 (41)	2 (29)
TIA	27 (11)	23 (13)	2 (5)	2 (7)	0 (0)
Peripheral arterial disease (*n* = 251)	18 (7)	10 (6)	4 (9)	3 (11)	1 (14)

BGC = basal ganglia calcification, TIA = transient ischaemic attack.

**Table 2:** Mean mobility outcomes stratified by BGC severity.

		Functional Mobility TUG (Seconds)	Balance (POMA-B)	Gait (POMA-G)	Overall Mobility (POMA-Total)	Recurrent Falls *n*/*N* (%) (Total 246)
Mean score total		14 (SD7.5)	12.1 (SD3.2)	9.3 (SD2.8)	21.3 ( SD5)	173/246 (70)
BGC severity	None	14.2 (SD8)	12.2 (SD3)	9.5 (SD3)	21.4 (SD5)	121/171 (71)
	Mild	12.6 (SD6)	12.4 (SD3)	9.3 (SD3)	21.8 (SD5)	29/40 (73)
	Moderate	15.6 (SD7)	11.7 (SD3)	8.3 (SD3)	19.9 (SD6)	18/28 (64)
	Severe	16.3 (SD8)	11.4 (SD5)	8.7 (SD3)	20.1 (SD7)	5/7 (71)

TUG = Timed Up and Go, BGC = basal ganglia calcification.

**Table 3:** Association between BGC presence and mobility outcomes.

	Model 1 * BGC, OR (95% CI)	*p*-Value	Model 2 BGC, OR (95% CI)	*p*-Value
Gait (POMA-G)	1.10 (0.63–1.92)	0.743	1.16 (0.64–2.01)	0.617
Balance (POMA-B)	0.99 (0.53–1.86)	0.972	1.03 (0.53–2.00)	0.927
Overall mobility (POMA total)	1.05 (0.58–1.89)	0.876	1.09 (0.59–2.03)	0.779
Functional mobility (TUG)	1.12 (0.56–2.27)	0.747	1.37 (0.65–2.91)	0.405
Recurrent falls	0.93 (0.52–1.69)	0.822	0.91 (0.50–1.66)	0.755

* Model 1: unadjusted analysis. Model 2: adjusted for age, sex, and history of cardiovascular disease.

## Data Availability

The datasets generated and/or analyzed during the current study are not publicly available due to privacy and ethical restrictions but are available from the corresponding author on reasonable request.
